# Supplementary material for: Distinct epigenetic programs regulate cardiac myocyte development and disease in the human heart in vivo
Source: Nat Commun. 2018 Jan 26;9:391. doi: 10.1038/s41467-017-02762-z (PMC5786002; doi:10.1038/s41467-017-02762-z)
Supplement: Supplementary file 1 — Supplementary Information [file 41467_2017_2762_MOESM1_ESM.pdf]

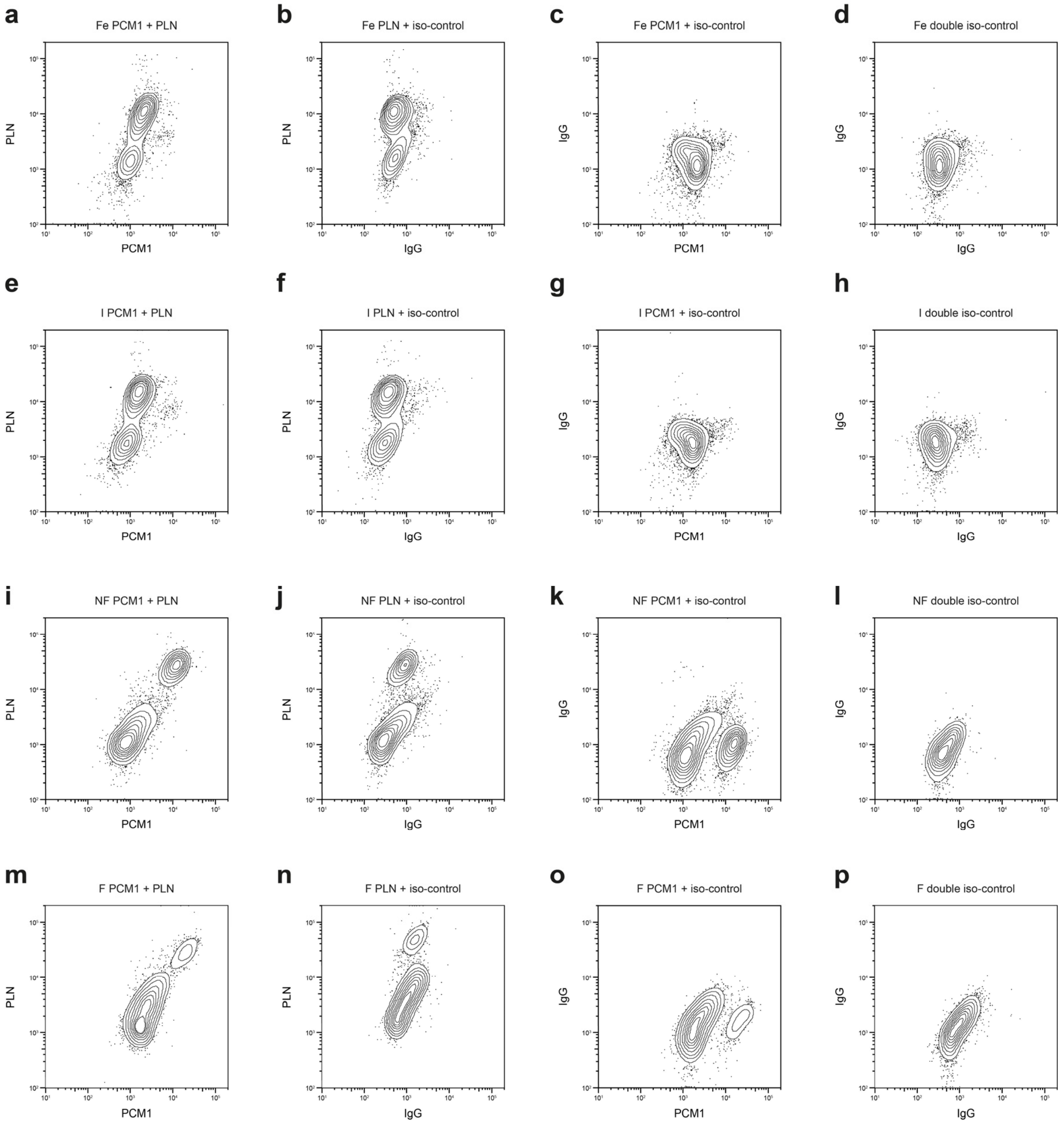

Supplementary Figure 1: Flow cytometry analysis of cardiac nuclei stained with antibodies against PCM1 and PLN or with IgG isotype controls.
(a-o) FACS diagrams of cardiac nuclei from n fetal **(a-d, ID 5)**, infantile **(e-h, ID 12)**, adult non-failing **(i-l, ID 14)** and adult failing **(m-p, ID 21)** heart labeled with PLN, PCM1 or isotype control (IgG) antibodies. IDs represent patient identities.

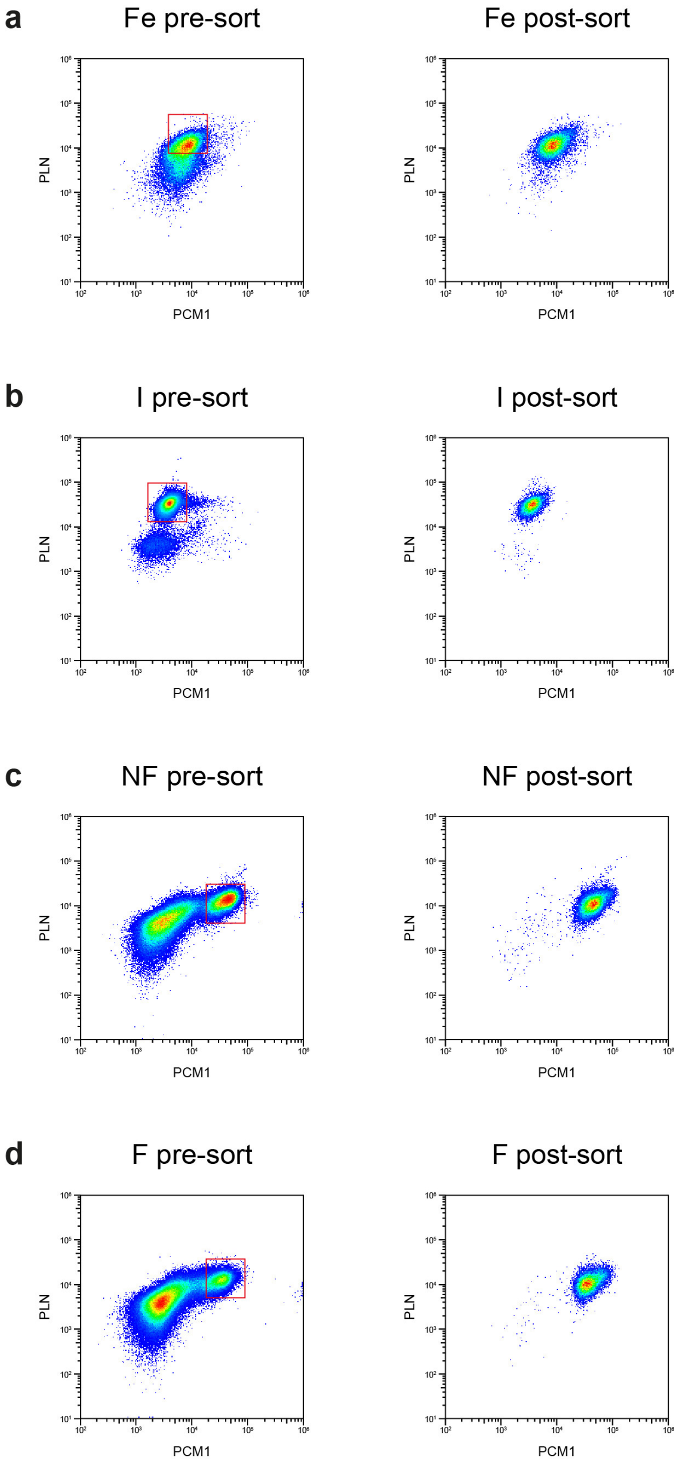

Supplementary Figure 2: Flow cytometric analysis of cardiac myocyte nuclei before (pre-sort) and after (post-sort) sorting

(a-d) Nuclei were labeled with antibodies against pericentriolar material 1 (PCM1) and phospholamban (PLN). FACS diagrams show representative results of fetal (a), infantile (b), adult non-failing (c) and failing cardiac nuclei (d). Red boxes indicate representative sorting gates.

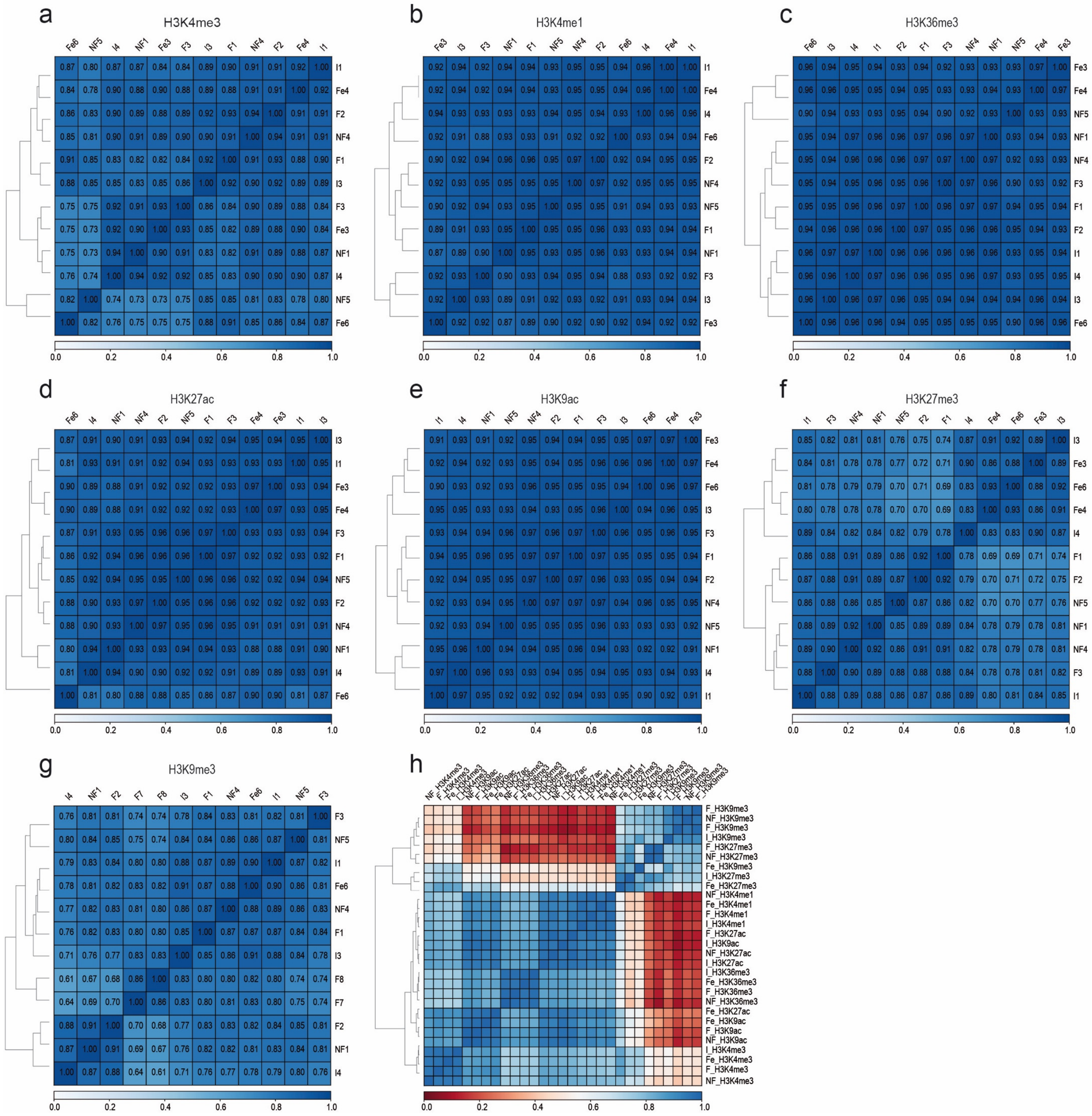

Supplementary Figure 3: Correlation of ChIP-seq data obtained from biological replicates. Heatmaps of Spearman correlation coefficients of ChIP-seq reads for the different histone modifications in genome-wide genic regions.

(a-g) Results obtained from biological replicates for different histone modifications.

(h) Data of biological replicates (n = 3) were merged and correlation coefficients were calculated for all assessed histone modifications and stages.

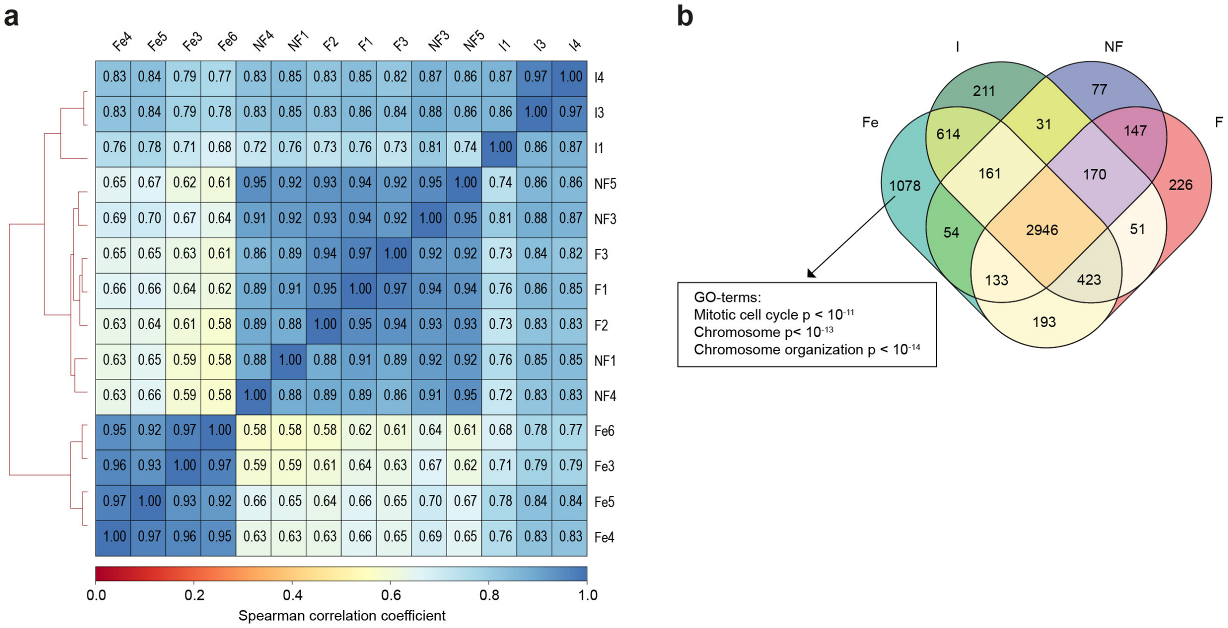

Supplementary Figure 4: Comparison of gene expression during development, maturation and disease.(a) Heatmap of Spearman correlation coefficients of RNA-seq reads obtained from biological replicates. Correlation coefficients were obtained for genes differentially regulated ($q \leq 0.05$, ≥ 2fold) between at least two stages.
(b) Venn diagram of genes with ≥ 1 FPKM (mean of n =3-4) in fetal (Fe), infant (I), adult non-failing (NF) or adult failing (F) cardiac myocytes.

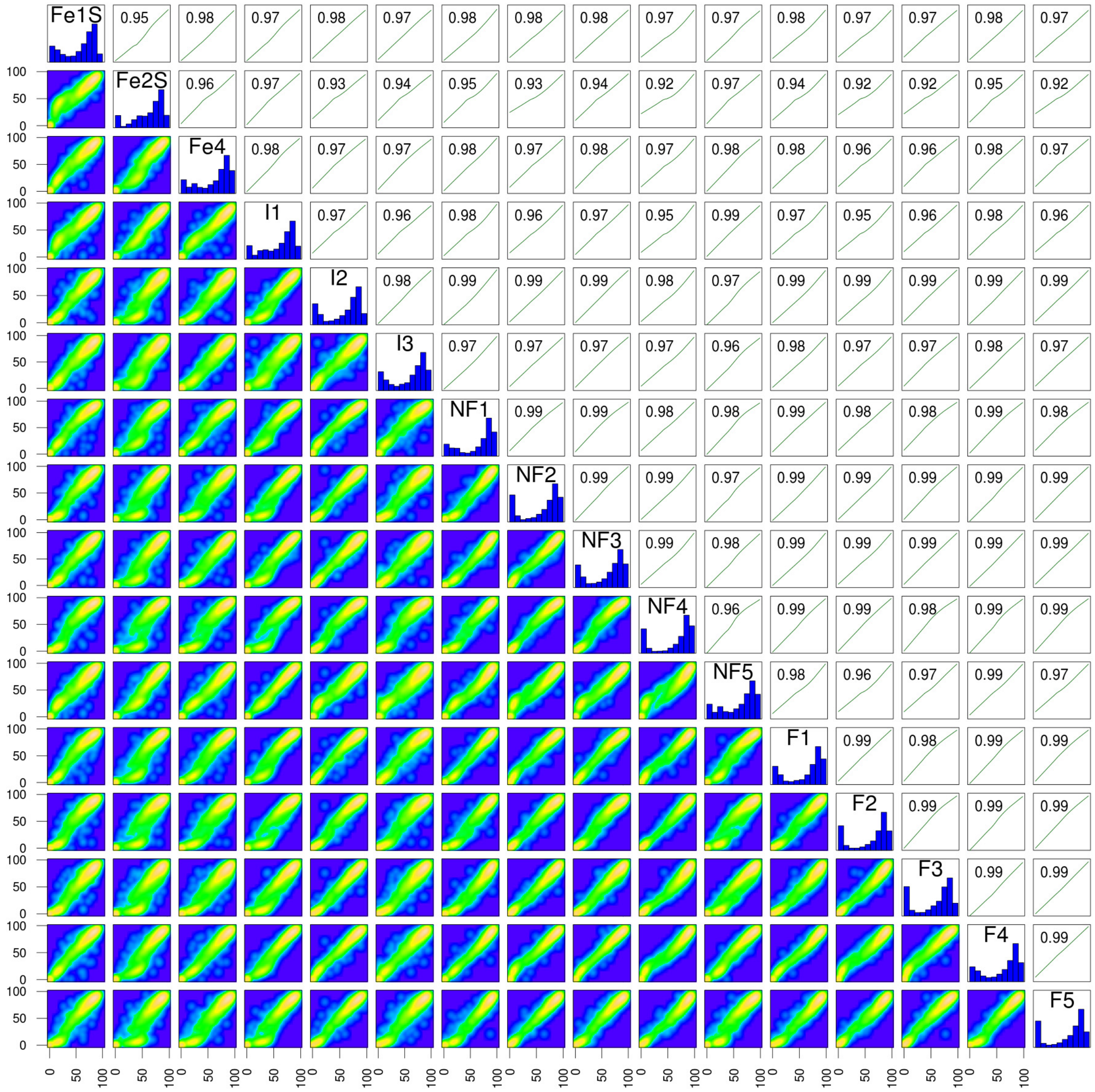

Supplementary Figure 5: Histograms of mCpG, scatter plots and Pearson correlation coefficients for all assessed replicates

Data represent CpGs with a minimal coverage of 20 in each replicate. DNA methylomes were generated from signal-regulatory protein alpha (SIRPA)-positive fetal cardiac myocytes (S) or cardiac myocyte nuclei identified by PCM1 and PLN staining. Shown are data from biological replicates of fetal (Fe), infantile (I), adult non-failing (NF) and adult failing cardiac myocyte nuclei (F). Numbers indicate Pearson correlation coefficients.

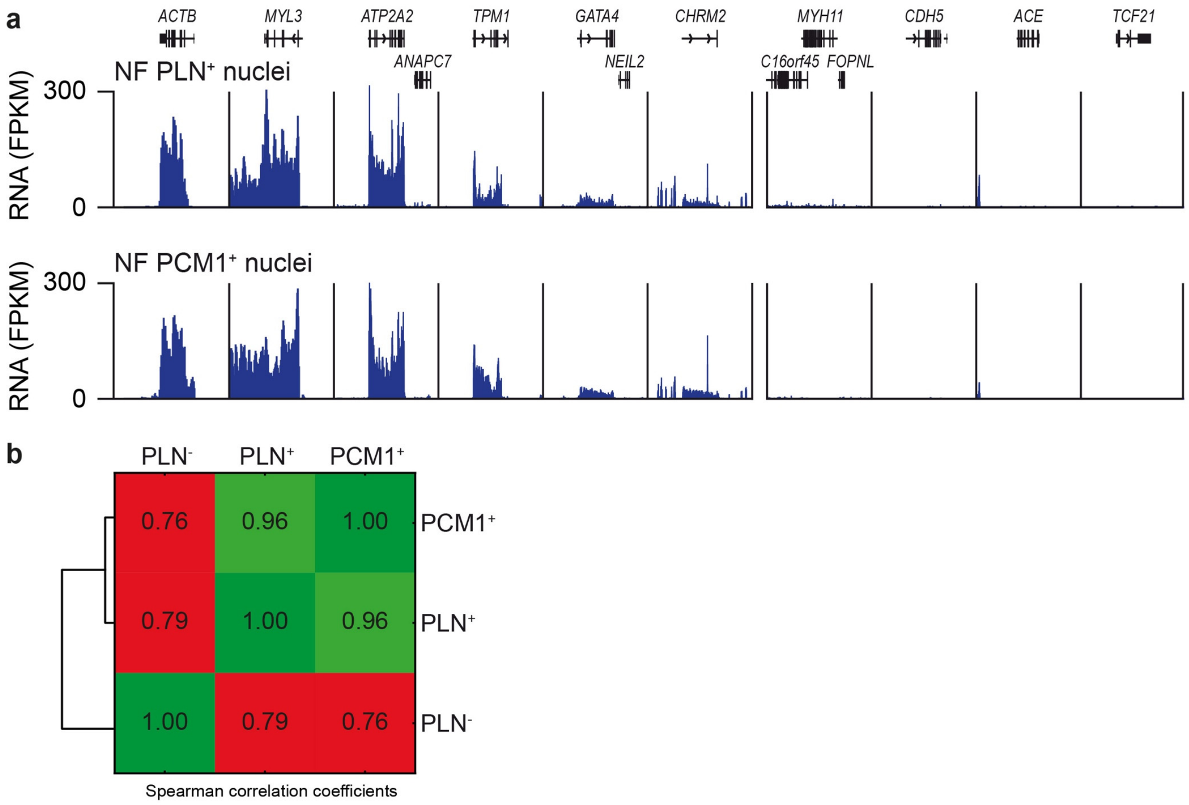

Supplementary Figure 6: RNA expression in cardiac myocyte nuclei sorted by PLN or PCM1 antibody staining

(a) Original traces of RNA-seq experiments from adult non-failing cardiac myocyte nuclei for different genes known to be expressed in cardiac myocytes (*ACTB, MYL3, ATP2A2, TPM1, GATA4, CHRM2*). In addition, marker genes for non-cardiac myocytes, including myosin heavy chain 11 (*MYH11*, smooth muscle cells), cadherin 5 and angiotensin converting enzyme (*CDH5*, *ACE*, endothelial cells) as well as transcription factor 21 (*TCF21*, adult mesenchymal cells) are shown.

(b) Heatmap of Spearman correlation coefficients of genome-wide distribution of RNA-seq reads obtained from PLN-negative, PLN-positive and PCM1-positive nuclei.

Abbreviations: *ACTB*, beta-actin; *MYL3*, myosin light chain 3; *ATP2A2*, sarcoplasmic reticulum calcium-ATPase 2; *TPM1*, tropomyosin 1; *GATA4*, GATA binding protein 4; *CHRM2*, muscarinic acetylcholine receptor M2.

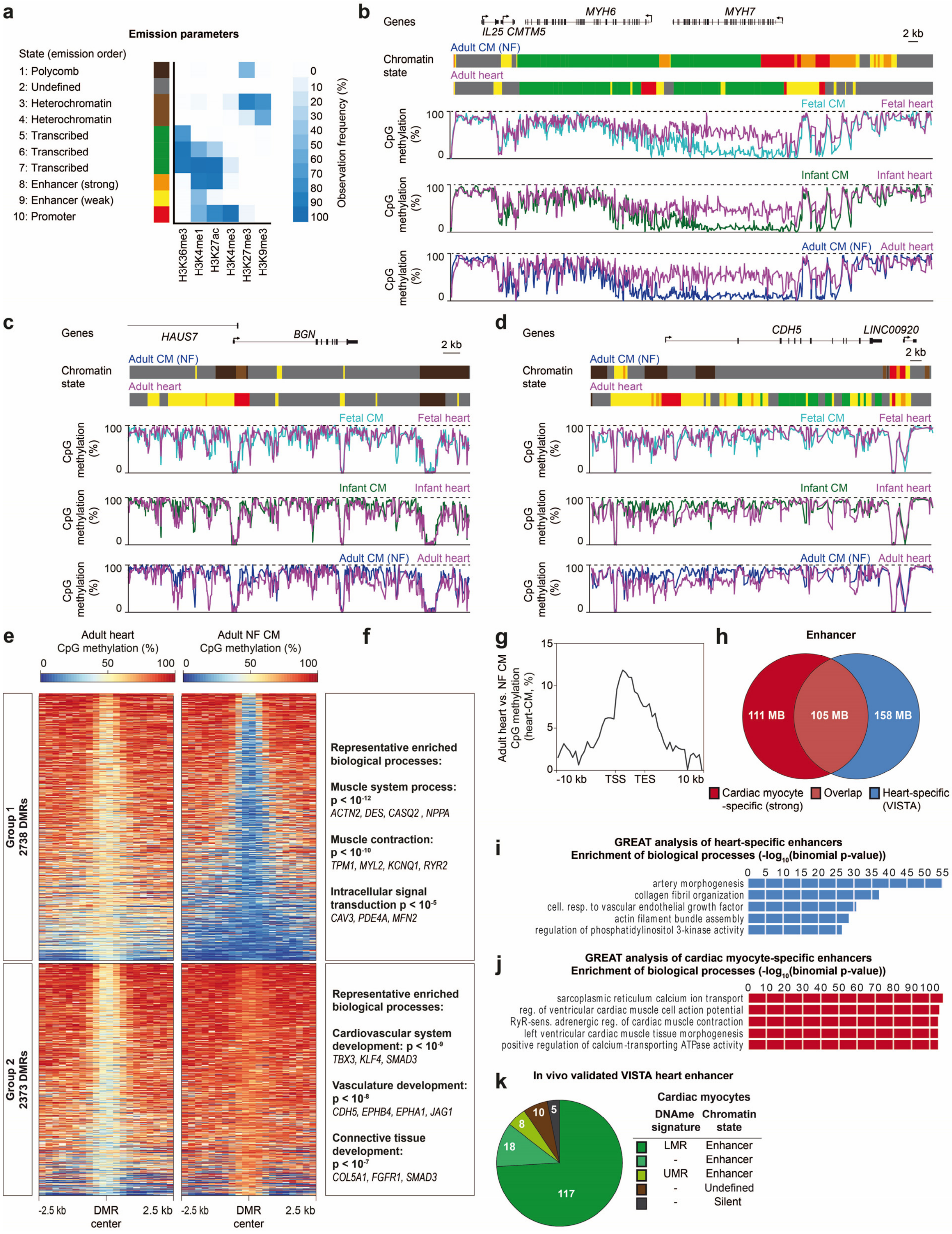

a
Emission parameters
State (emission order)
1: Polycomb
2: Undefined
3: Heterochromatin
4: Heterochromatin
5: Transcribed
6: Transcribed
7: Transcribed
8: Enhancer (strong)
9: Enhancer (weak)
10: Promoter
H3K36me3 H3K4me1 H3K27ac H3K4me3 H3K27me3 H3K9me3
Observation frequency (%)
0 10 20 30 40 50 60 70 80 90 100
b
Genes
MYH6
MYH7
IL25 CMTM5
2 kb
Chromatin state
Adult CM (NF)
Adult heart
CpG methylation (%)
Fetal CM
Fetal heart
Infant CM
Infant heart
Adult CM (NF)
Adult heart
c
Genes
HAUS7
BGN
2 kb
d
Genes
CDH5
LINC00920
2 kb
e
Adult heart CpG methylation (%)
Adult NF CM CpG methylation (%)
0 50 100
Group 1 2738 DMRs
Group 2 2373 DMRs
-2.5 kb
DMR center
2.5 kb
f
Representative enriched biological processes:
Muscle system process: p < 10^-12
ACTN2, DES, CASQ2 , NPPA
Muscle contraction: p < 10^-10
TPM1, MYL2, KCNQ1, RYR2
Intracellular signal transduction p < 10^-5
CAV3, PDE4A, MFN2
Representative enriched biological processes:
Cardiovascular system development: p < 10^-9
TBX3, KLF4, SMAD3
Vasculature development: p < 10^-8
CDH5, EPHB4, EPHA1, JAG1
Connective tissue development: p < 10^-7
COL5A1, FGFR1, SMAD3
g
Adult heart vs. NF CM CpG methylation (heart-CM, %)
0 5 10 15
-10 kb TSS TES 10 kb
h
Enhancer
111 MB
105 MB
158 MB
Cardiac myocyte -specific (strong)
Overlap
Heart-specific (VISTA)
i
GREAT analysis of heart-specific enhancers
Enrichment of biological processes (-log10(binomial p-value))
0 5 10 15 20 25 30 35 40 45 50 55
artery morphogenesis
collagen fibril organization
cell. resp. to vascular endothelial growth factor
actin filament bundle assembly
regulation of phosphatidylinositol 3-kinase activity
j
GREAT analysis of cardiac myocyte-specific enhancers
Enrichment of biological processes (-log10(binomial p-value))
0 10 20 30 40 50 60 70 80 90 100
sarcoplasmic reticulum calcium ion transport
reg. of ventricular cardiac muscle cell action potential
RyR-sens. adrenergic reg. of cardiac muscle contraction
left ventricular cardiac muscle tissue morphogenesis
positive regulation of calcium-transporting ATPase activity
k
In vivo validated VISTA heart enhancer
117
18
8
10
5
Cardiac myocytes
DNAme signature
Chromatin state
LMR Enhancer
- Enhancer
UMR Enhancer
- Undefined
- Silent

Supplementary Figure 7: Comparison of cardiac myocyte and heart epigenome data
(a) Chromatin state model derived from adult cardiac myocytes.
(b-d) Original traces of mCpG in fetal, infantile and adult heart tissue (purple traces) compared with the respective cardiac myocyte data. Shown are loci containing the cardiac myocyte marker genes alpha and beta myosin heavy chain (*MYH6, MYH7*), the fibroblast gene biglycan (*BGN*) as well as the endothelial cell marker gene VE-cadherin (*CDH5)*. Chromatin states of adult heart and cardiac myocytes are annotated.
(e) Heatmaps of differentially CpG-methylated regions between adult heart and cardiac myocytes.
(f) Gene ontology analysis of genes overlapping differentially methylated regions in hearts and cardiac myocytes. Depicted are representative GO terms and genes.
(g) Differential mCpG in heart tissue versus adult NF CM at flanking regions of highly expressed genes (mean >100 FPKM in adult NF CM).
(h) Overlap of strong enhancers identified in cardiac myocyte nuclei by ChromHMM with predicted VISTA heart enhancers[23].
(i, j) GREAT analysis of enhancer regions detected specifically in heart tissue (**j,** heart-specific) or cardiac myocytes (**j,** cardiac myocyte-specific).
(k) Overlap of *in vivo* confirmed VISTA heart enhancers[23] with hypomethylated (LMR, UMR) regions as well as the associated chromatin states.
Figures show cardiac myocyte mCpG data from 3-5 biological replicates and heart tissue mCpG data from 1 biological replicate.

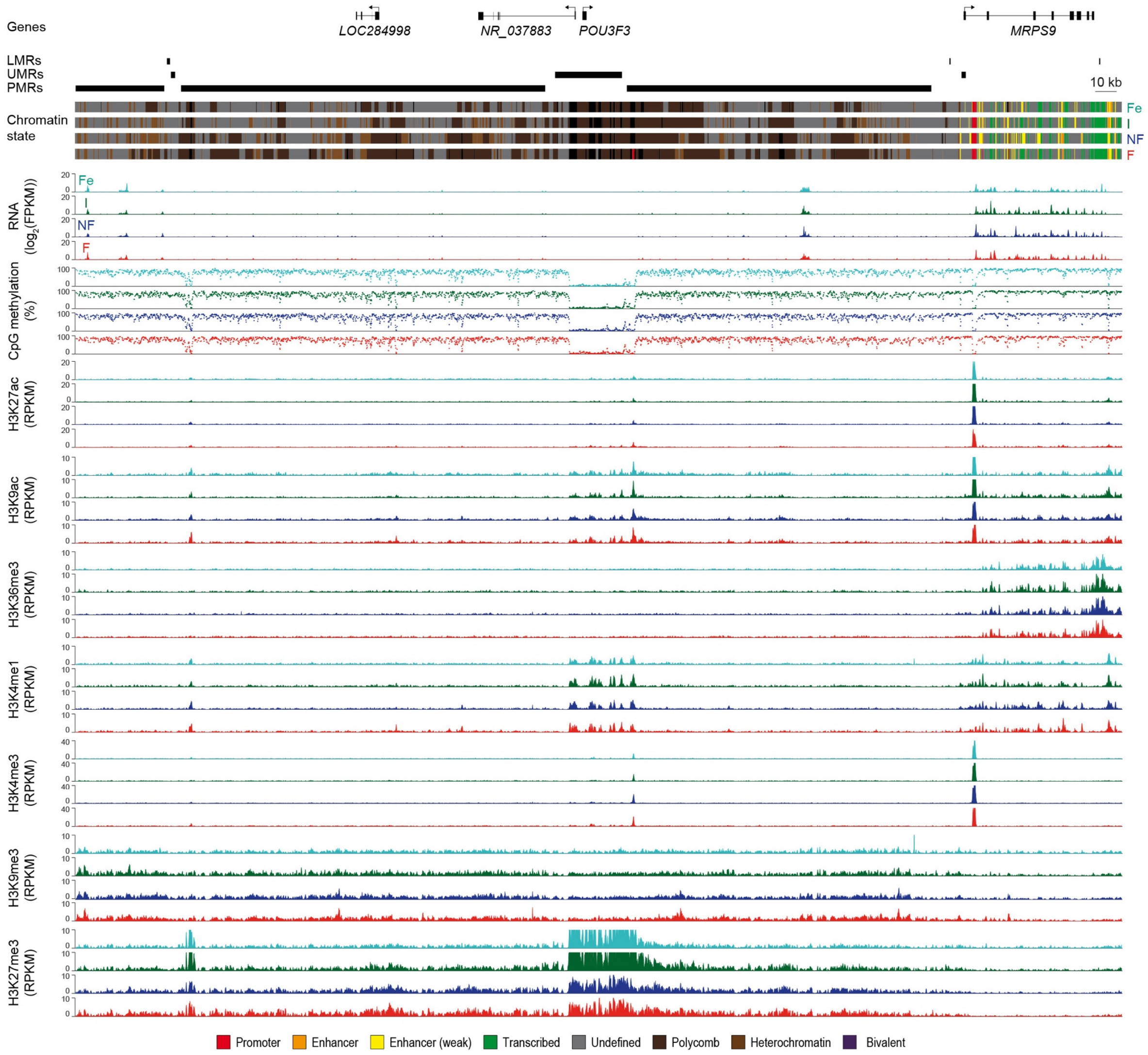

Supplementary Figure 8: Genomic region of the *POU3F3* gene with annotation of mCpG-guided genome segmentation

Original traces of RNA expression, mCpG and histone marks of the POU class 3 homeobox 3 (*POU3F3*) gene region in fetal (Fe), infantile (I), adult non-failing (NF) and failing (F) cardiac myocytes. Low methylated regions (LMR), unmethylated regions (UMR) and partially methylated regions (PMR) and chromatin states are annotated. Traces show data from n biological replicates: mCpG, n = 3-5; H3K27ac, H3K9ac, H3K36me3, H3K4me1, H3K4me3, H3K27me3 and H3K9me3, n = 3; RNA-seq n= 3-4.

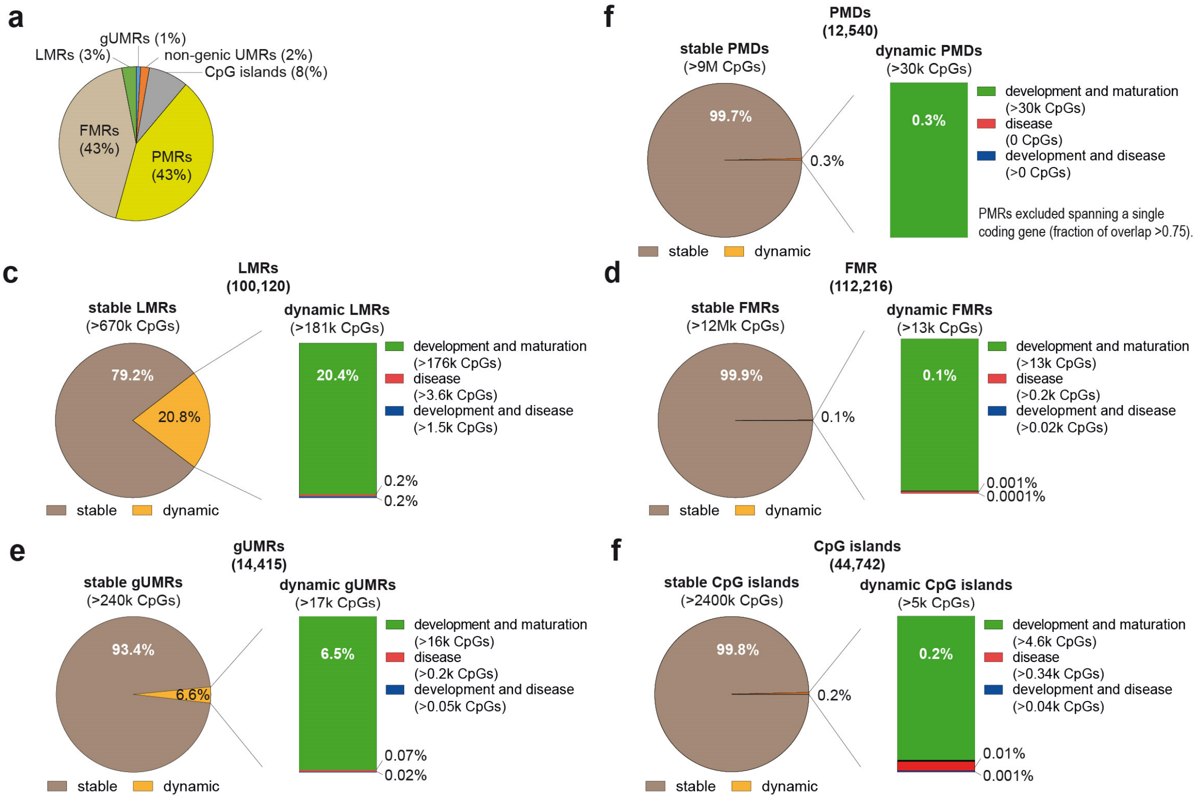

Supplementary Figure 9: mCpG-guided genome segmentation

(a) Genome-wide distribution of CpGs in annotated genomic regions. Abbreviations: LMR, low methylated region; UMR, unmethylated region; gUMR, genic UMR; PMR, partially methylated region; FMR, fully methylated region.

(b-f) Classification of PMRs (b), LMRs (c), FMRs (d), gUMRs (e), and CpG islands (f) into stable (no change in mean mCpG levels between fetal, infantile, adult non-failing and failing cardiac myocytes) or dynamic regions (significant changes in mCpG levels between fetal, infantile and adult samples). Percent values represent the proportion of affected regions.

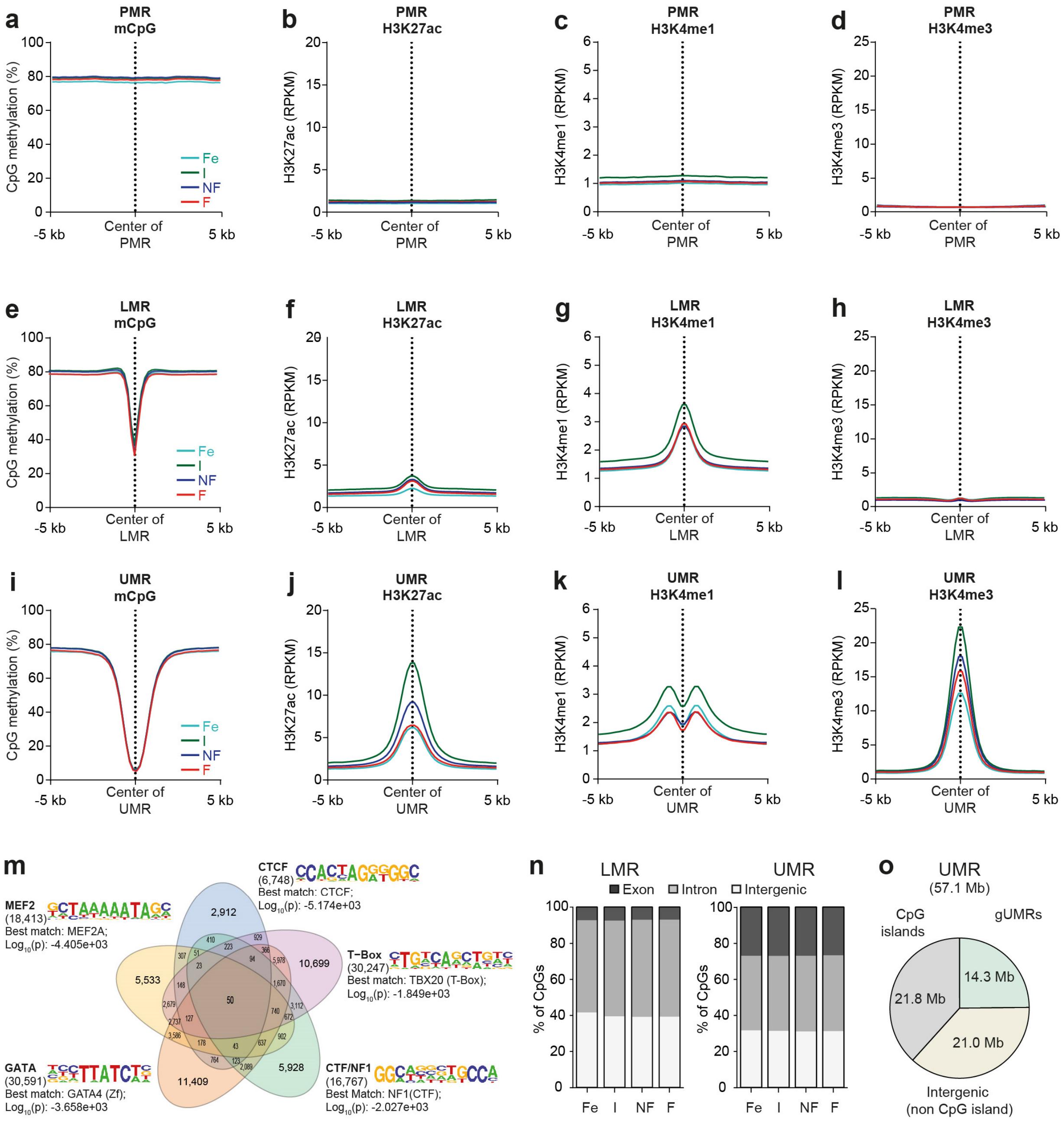

Supplementary Figure 10: Annotation of functional genomic elements by mCpG-guided genome segmentation

(a-l) Average plots of mCpG and histone enrichments are shown for PMRs (a-d), LMRs (e-h), and UMRs (i-l) for fetal, infant, adult non-failing and failing cardiac myocytes.

(m) Transcription factor motifs in LMRs. Given is the number of transcription factor motifs and the HOMER transcription factor enrichment p-value.

(n) Mapping of LMRs and UMRs to exon, intron and intergenic regions.

(o) Genome-wide overlap of UMRs with CpG islands and genic UMRs.

Figures show data from n biological replicates: mCpG, n = 3-5; H3K27ac, H3K9ac, H3K36me3, H3K4me1, H3K4me3, H3K27me3 and H3K9me3, n = 3).

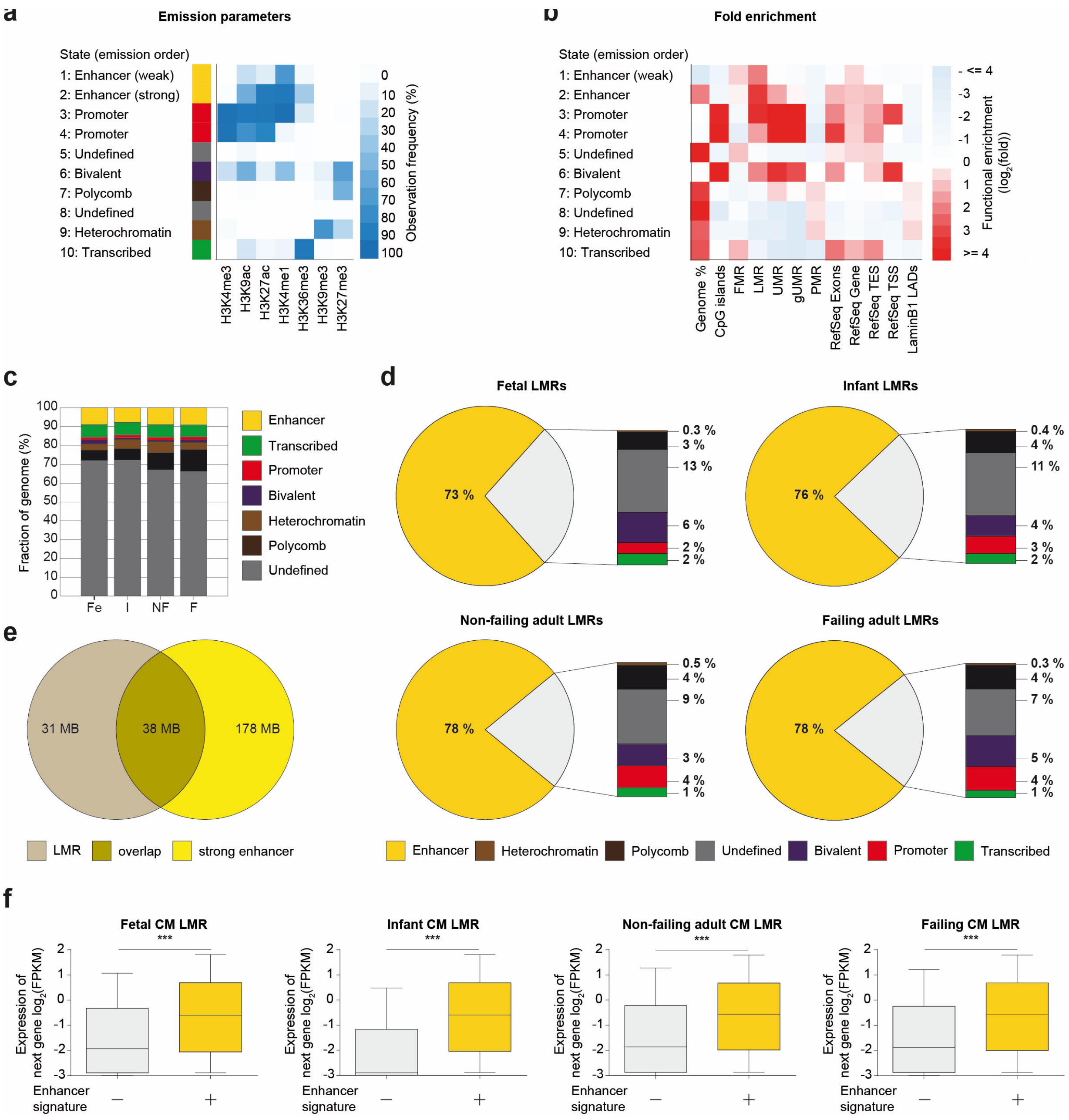
a
Emission parameters
State (emission order)
1: Enhancer (weak)
2: Enhancer (strong)
3: Promoter
4: Promoter
5: Undefined
6: Bivalent
7: Polycomb
8: Undefined
9: Heterochromatin
10: Transcribed
H3K4me3
H3K9ac
H3K27ac
H3K4me1
H3K36me3
H3K9me3
H3K27me3
Observation frequency (%)
b
Fold enrichment
State (emission order)
1: Enhancer (weak)
2: Enhancer
3: Promoter
4: Promoter
5: Undefined
6: Bivalent
7: Polycomb
8: Undefined
9: Heterochromatin
10: Transcribed
Genome %
CpG islands
FMR
LMR
UMR
gUMR
PMR
RefSeq Exons
RefSeq Gene
RefSeq TES
RefSeq TSS
LaminB1 LADs
Functional enrichment (log2(fold))
c
Fraction of genome (%)
Fe
I
NF
F
Enhancer
Transcribed
Promoter
Bivalent
Heterochromatin
Polycomb
Undefined
d
Fetal LMRs
73 %
0.3 %
3 %
13 %
6 %
2 %
2 %
Infant LMRs
76 %
0.4 %
4 %
11 %
4 %
3 %
2 %
Non-failing adult LMRs
78 %
0.5 %
4 %
9 %
3 %
4 %
1 %
Failing adult LMRs
78 %
0.3 %
4 %
7 %
5 %
4 %
1 %
Enhancer
Heterochromatin
Polycomb
Undefined
Bivalent
Promoter
Transcribed
e
31 MB
38 MB
178 MB
LMR
overlap
strong enhancer
f
Fetal CM LMR
Infant CM LMR
Non-failing adult CM LMR
Failing CM LMR
***
Expression of next gene log2(FPKM)
Enhancer signature
–
+

Supplementary Figure 11: Chromatin state of LMRs.
(a, b) Heat maps for chromatin states generated by ChromHMM.
a) Chromatin states were learned using a multi variant hidden Markov model on the basis of genome-wide occurrence of combinations of different histone marks in non-failing adult cardiac myocytes.
(b) The enrichment of chromatin states in CpG islands, fully methylated regions (FMR), low methylated regions (LMR), unmethylated regions (UMR), genic UMRs (gUMR), partially methylated domains (PMR), and Refseq Exons, genes, transcription end (TES) and start sites (TSS) as well as lamina-associated domains (LADs) are shown for adult non-failing cardiac myocytes.
(c) Genomic proportion of the chromatin states.
(d) Chromatin state of annotated LMRs.
(e) Overlap of LMRs detected in CM and strong ChromHMM enhancers.
(f) Expression of genes adjacent to LMRs with and without enhancer signature. Shown are boxplots with 10-90% whiskers. *** $p < 0.001$, t-test

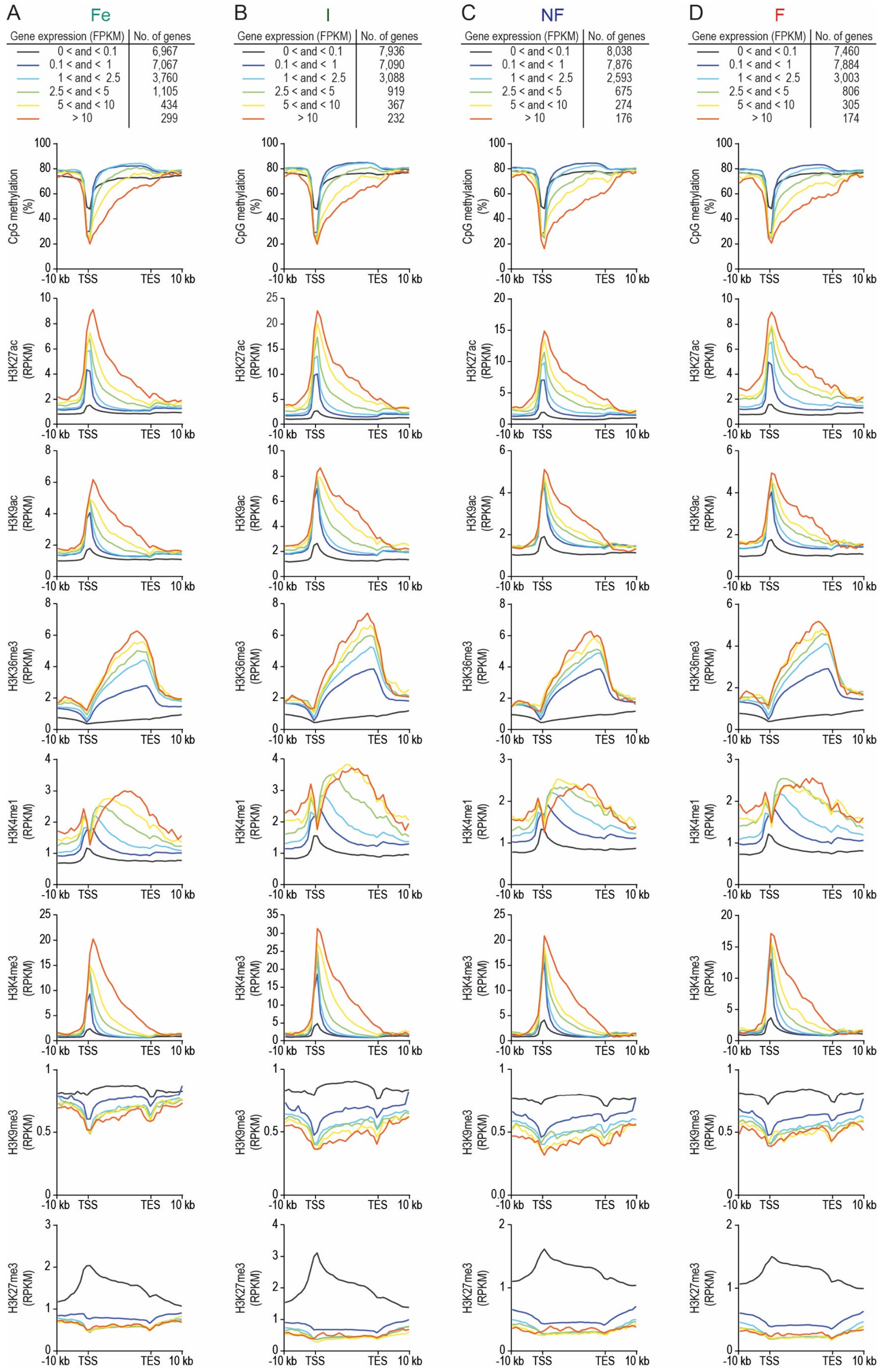
A
Fe
Gene expression (FPKM) | No. of genes
0 < and < 0.1 6,967
0.1 < and < 1 7,067
1 < and < 2.5 3,760
2.5 < and < 5 1,105
5 < and < 10 434
> 10 299
B
I
Gene expression (FPKM) | No. of genes
0 < and < 0.1 7,936
0.1 < and < 1 7,090
1 < and < 2.5 3,088
2.5 < and < 5 919
5 < and < 10 367
> 10 232
C
NF
Gene expression (FPKM) | No. of genes
0 < and < 0.1 8,038
0.1 < and < 1 7,876
1 < and < 2.5 2,593
2.5 < and < 5 675
5 < and < 10 274
> 10 176
D
F
Gene expression (FPKM) | No. of genes
0 < and < 0.1 7,460
0.1 < and < 1 7,884
1 < and < 2.5 3,003
2.5 < and < 5 806
5 < and < 10 305
> 10 174
CpG methylation (%)
H3K27ac (RPKM)
H3K9ac (RPKM)
H3K36me3 (RPKM)
H3K4me1 (RPKM)
H3K4me3 (RPKM)
H3K9me3 (RPKM)
H3K27me3 (RPKM)
-10 kb TSS TES 10 kb

Supplementary Figure 12: Profiles of mean mCpG and histone modifications of genes grouped according to their expression level.

(a-d) Results for cardiac myocyte nuclei from fetal (a), infantile (b), adult non-failing (c) and failing (d) hearts. Tables summarize the number of genes for the different RNA expression groups. Plots represent genic regions from TSS (transcription start site) to TES (transcription end site) and 10 kb flanking regions for mCpG and histone marks. For ease of comparison, graphs for adult non-failing hearts displayed in Fig. 2a are shown in this figure, too (c).

Figures show data from n biological replicates: mCpG, n = 3-5; H3K27ac, H3K9ac, H3K36me3, H3K4me1, H3K4me3, H3K27me3 and H3K9me3, n = 3.

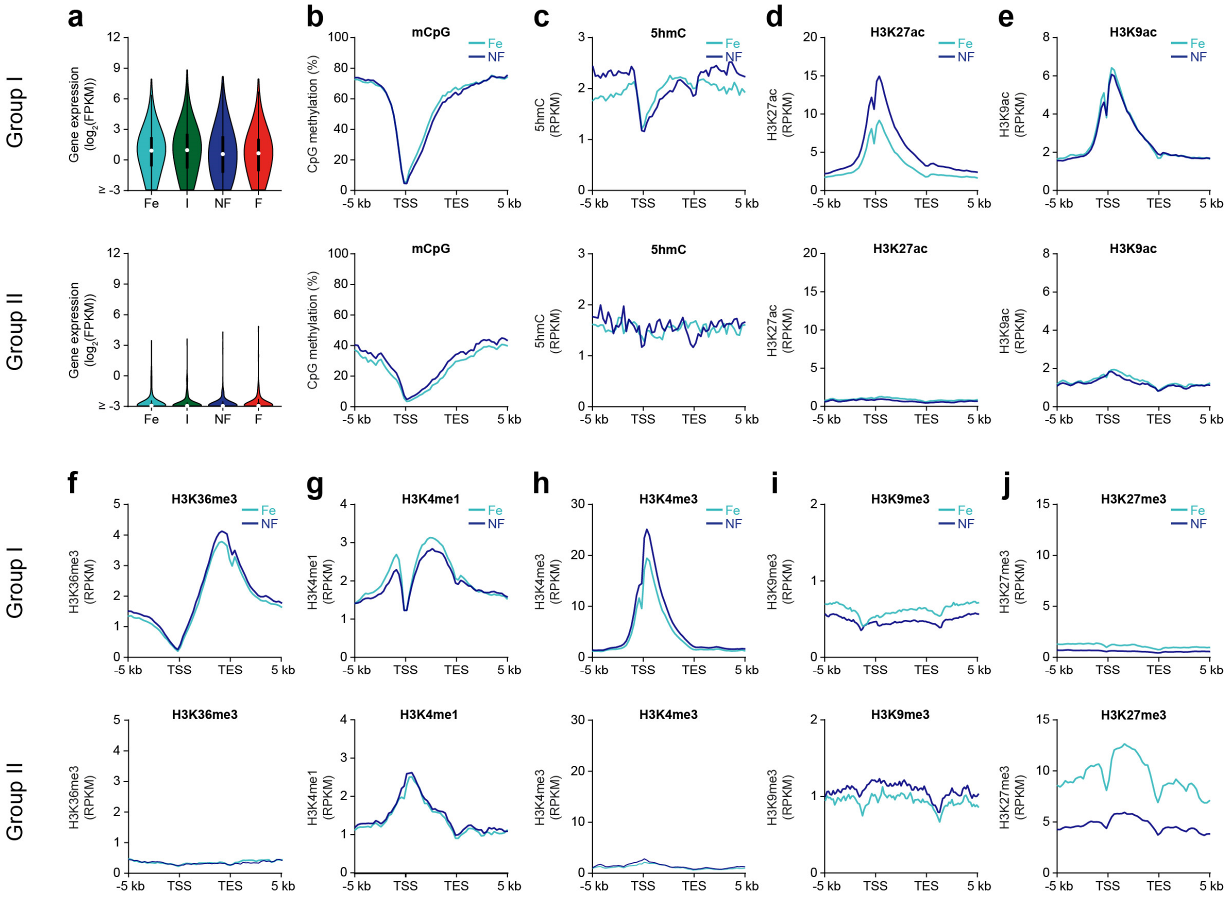

Supplementary Figure 13: RNA expression, mCpG and histones marks of genes with gUMR (a-j) Gene expression values (a), profiles of mCpG (b) and 5-hydroxymethylcytosine (5hmC) levels (c) as well as enrichment of histone modifications (d-j) in genes with distinct low genic methylation. Genes were clustered as shown in Fig. 2b.

Figures show data from n biological replicates: mCpG, n = 3-5; H3K27ac, H3K9ac, H3K36me3, H3K4me1, H3K4me3, H3K27me3 and H3K9me3, n = 3; RNA, n= 3-4

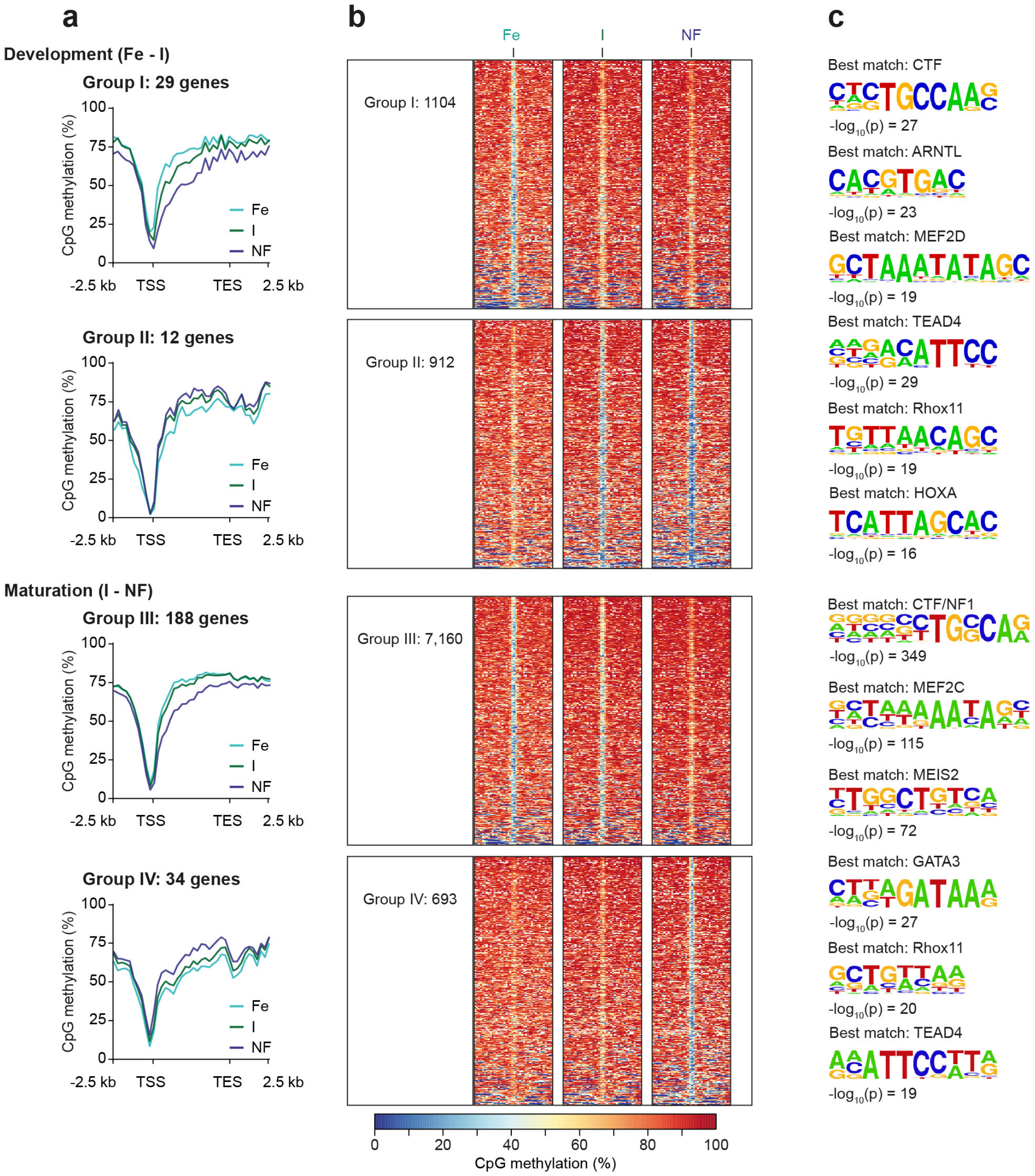

Supplementary Figure 14: mCpG of low methylated and genic unmethylated regions (LMR, gUMR) during development of fetal and maturation of infantile cardiac myocytes.
(a-c) Analysis of differential mCpG during development of fetal (Fe) to infantile (I) cardiac myocytes (group I, II) or during maturation of infantile (I) to adult (NF) cardiac myocytes (group III, IV). Group I and III represent regions with loss and II and IV with gain of mCpG. Shown are data of genes with differential gUMR (a) and LMR (b) mCpG as well as of enriched transcription factor motifs in LMRs (c). Shown are 10kb windows around the LMR center. HOMER transcription factor enrichment p-values are given.
Figures show data from n biological replicates: mCpG, n = 3-5

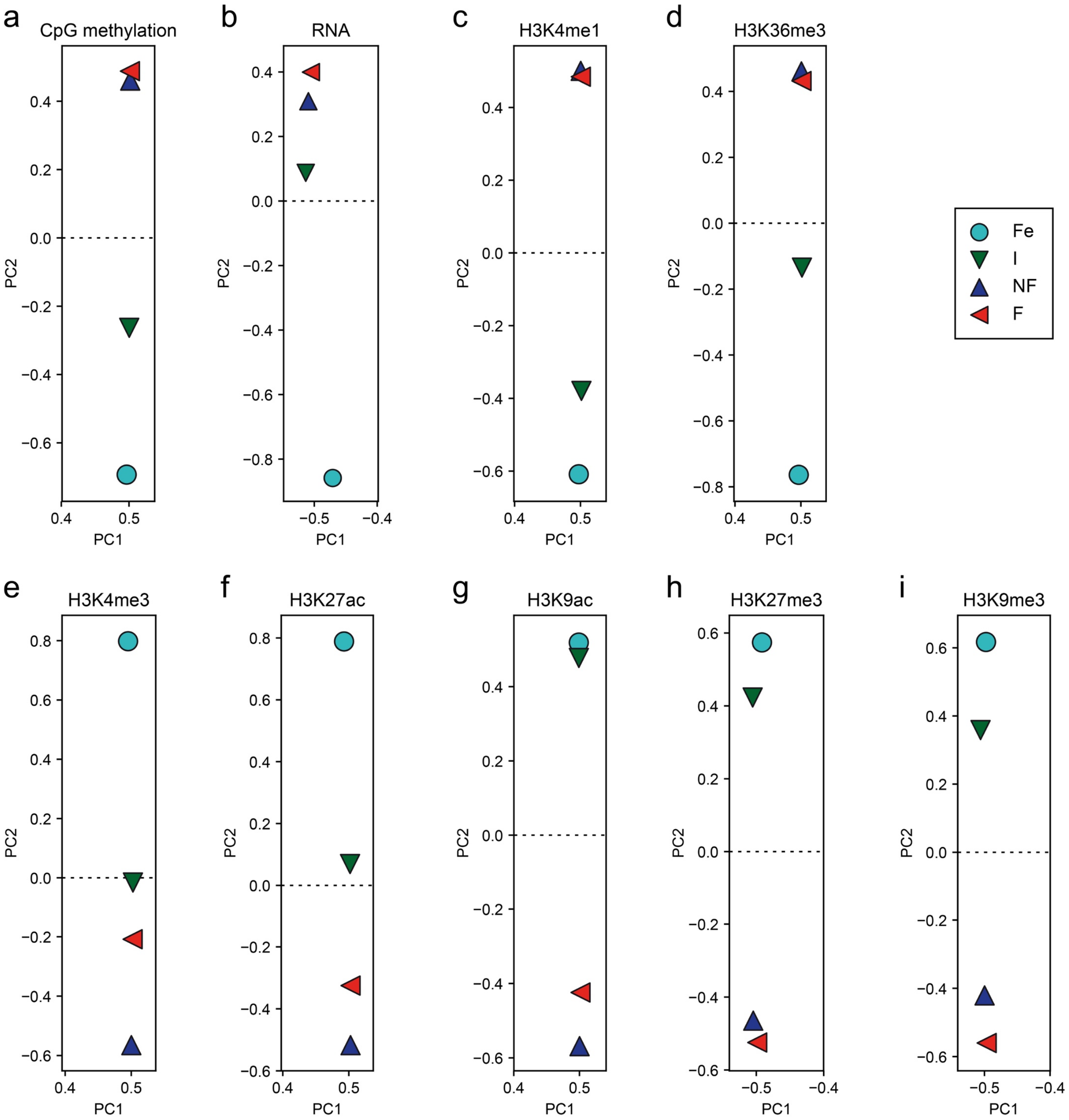

Supplementary Figure 15: Prinicipal component analysis
Principal component analysis of WGBS **(a)**, RNA-seq **(b)** and ChIP-seq **(c-i)** data of genic regions. Figures show data from n biological replicates: mCpG, n = 3-5; H3K27ac, H3K9ac, H3K36me3, H3K4me1, H3K4me3, H3K27me3 and H3K9me3, n = 3; RNA, n= 3-4

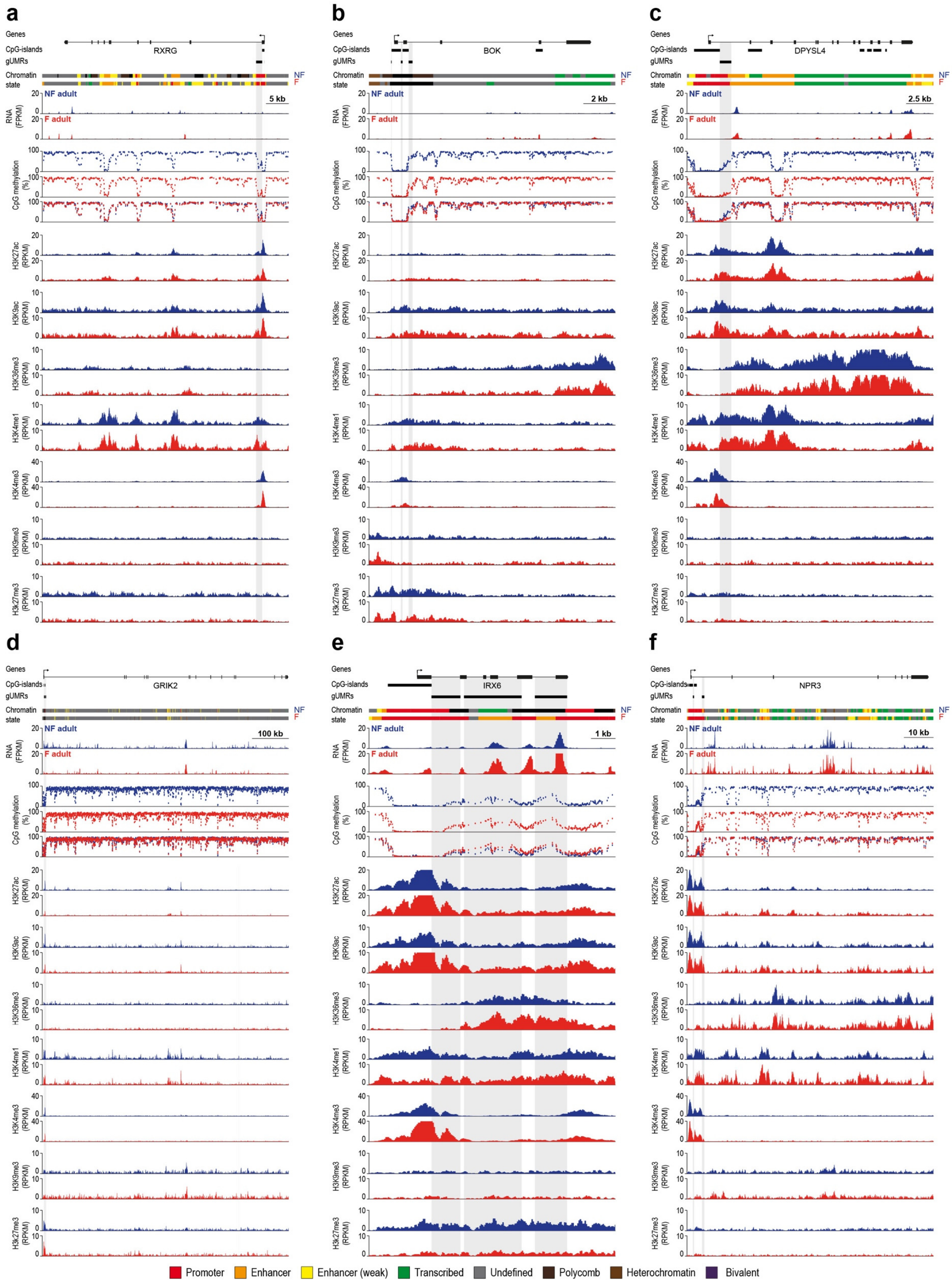
a
b
c
d
e
f
Genes
CpG-islands
gUMRs
Chromatin state
NF
F
RXRG
BOK
DPYSL4
GRIK2
IRX6
NPR3
NF adult
F adult
5 kb
2 kb
2.5 kb
100 kb
1 kb
10 kb
RNA (FPKM)
CpG methylation (%)
H3K27ac (RPKM)
H3K9ac (RPKM)
H3K36me3 (RPKM)
H3K4me1 (RPKM)
H3K4me3 (RPKM)
H3K9me3 (RPKM)
H3K27me3 (RPKM)
Promoter
Enhancer
Enhancer (weak)
Transcribed
Undefined
Polycomb
Heterochromatin
Bivalent

Supplementary Figure 16: Original traces of genes with differential CpG of the gene body in failing *vs.* non-failing cardiac myocytes

(a-f) RNA-seq, mCpG, histone ChIP-seq traces and chromatin state are displayed for genes with differential gUMR methylation in failing cardiac myocytes compared to non-failing cardiac myocytes.

Figures show data from n biological replicates: mCpG, n = 5; H3K27ac, H3K9ac, H3K36me3, H3K4me1, H3K4me3, H3K27me3 and H3K9me3, n = 3; RNA, n= 3-4

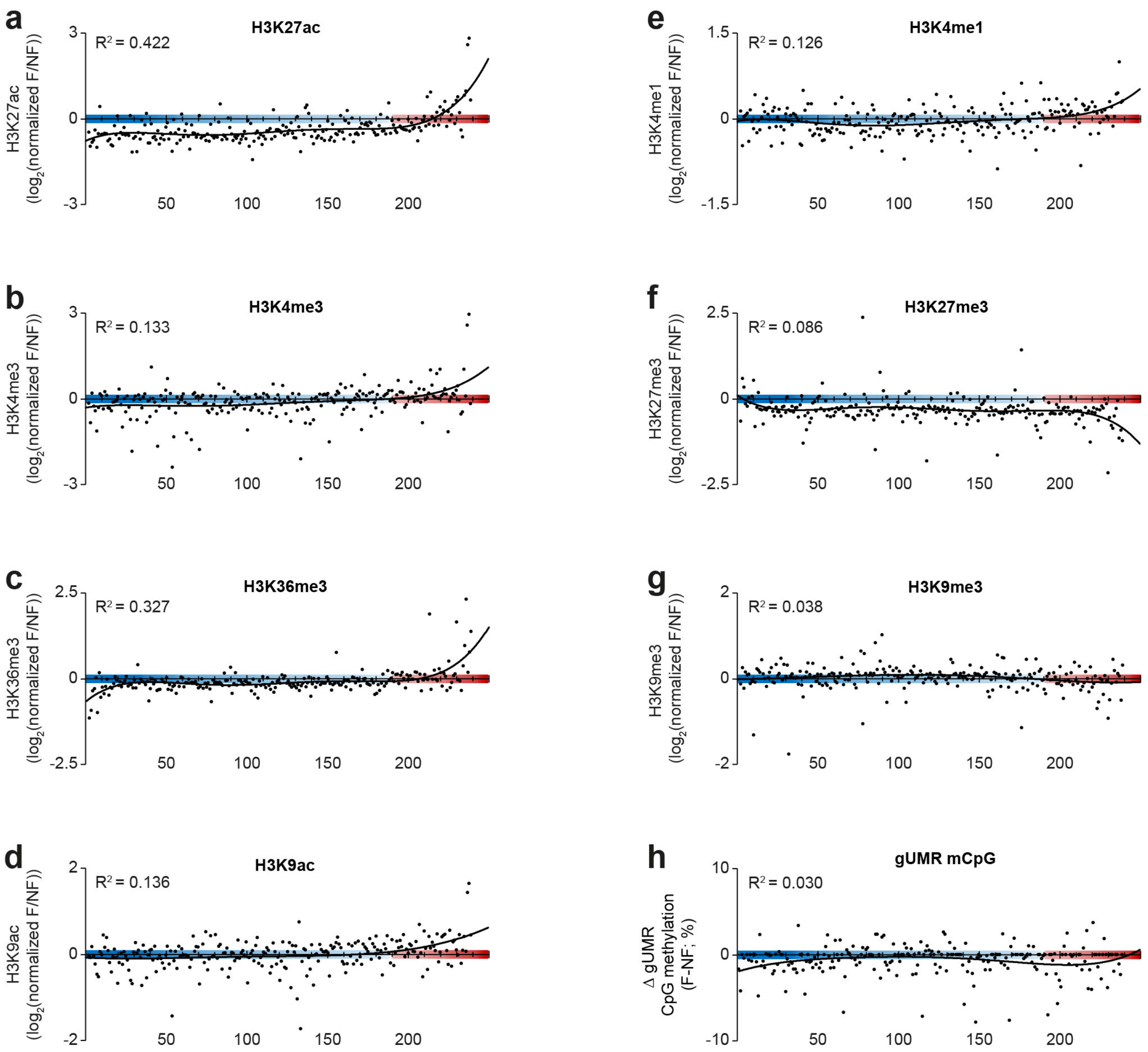

Supplementary Figure 17: mCpG and histone profiles of genes which are differentially expressed in failing cardiac myocytes.
Differently expressed genes in cardiac myocyte nuclei of failing *versus* non-failing hearts were ranked according to expression changes (x-axis). The relative enrichment of histone modifications (a-g) or changes in gUMR mCpG (h) are shown for the ranked genes. Resulting R^2-values of a non-linear regression (black line) are given.
Figures show data from n biological replicates: mCpG, n = 5; H3K27ac, H3K9ac, H3K36me3, H3K4me1, H3K4me3, H3K27me3 and H3K9me3, n = 3

Antibody	Manufacturer	ID	Amount per ChIP
anti-H3K27ac	Abcam	ab4729	4 µl
anti-H3K9ac	Active motif	39137	5 µl
anti-H3K36me3	Abcam	ab9050	4 µl
anti-H3K4me1	Abcam	ab8895	4 µl
anti-H3K4me3	Diagenode	pAb-003-050	4 µl
anti-H3K9me3	Diagenode	pAb193-050	1.5 µl
anti-H3K27me3	Merck Millipore	07-449	4 µl

Supplementary Table 1: Antibodies used for ChIP-seq
