# Supplementary material for: Distinct epigenetic programs regulate cardiac myocyte development and disease in the human heart in vivo
Source: Nat Commun. 2018 Jan 26;9:391. doi: 10.1038/s41467-017-02762-z (PMC5786002; doi:10.1038/s41467-017-02762-z)
Supplement: Supplementary file 3 — Description of Additional Supplementary Files [file 41467_2017_2762_MOESM3_ESM.pdf]

Description of Additional Supplementary Files

File Name: Supplementary Data 1
Description: Patient characteristics.

File Name: Supplementary Data 2
Description: Sequencing statistics.

File Name: Supplementary Data 3
Description: Genes with pronounced genic mCpG and GO analysis.

File Name: Supplementary Data 4
Description: LMRs with differential mCpG in fetal and adult non-failing cardiac myocytes.
